# P38 MAPK is involved in epigenetic regulation of fibrotic genes in replication induced senescence in lung fibroblasts

**DOI:** 10.18632/aging.206357

**Published:** 2026-03-03

**Authors:** Shan Zhu, Jennifer Q Zhou, Kan Wang, Ming-lei Guo, Yan Y Sanders

**Affiliations:** 1 Department of Biomedical and Translational Sciences, Eastern Virginia Medical School, Macon & Joan Brock Virginia Health Sciences at Old Dominion University, Norfolk 23510, VA, USA

**Keywords:** senescence, fibroblast activation, p38 MAPK, lung fibrosis, H4K16Ac

## Abstract

Fibroblast activation is essential for tissue repair following injury; however, prolonged activation drives pathological fibrosis. Idiopathic pulmonary fibrosis (IPF), a progressive and age-associated lung disease, is characterized by aberrant fibroblast activation, with increasing evidence implicating senescent and near-senescent fibroblasts in its pathogenesis. However, the underlying mechanisms remain poorly defined. In this study, we investigated whether histone modification is involved in TGF-β1 treated lung fibroblasts and contributes to the fibrotic phenotype. Human IMR90 lung fibroblasts at low and high population doubling levels (LPDL and HPDL), as well as primary IPF fibroblasts, were used in this study. In response to TGF-β1, both LPDL and HPDL fibroblasts upregulated profibrotic genes, including α-smooth muscle actin (α-SMA) and Collagen type III alpha 1 (Col3A1). Compared with LPDL fibroblasts, HPDL fibroblasts exhibited a delayed and sustained p38 MAPK response. Pharmacological inhibition of p38 MAPK significantly reduced α-SMA and Col3A1 expression in both TGF-β1-stimulated fibroblasts and primary IPF cells. Mechanistically, TGF-β1-induced expression of α-SMA and Col3A1 was mediated by histone H4K16 acetylation (H4K16ac), which was enriched at gene promoter regions and attenuated by p38 MAPK inhibition. These findings suggest that a p38 MAPK–dependent epigenetic mechanism is involved in fibroblast activation, supporting the therapeutic potential of p38 MAPK inhibition for treating age-related fibrotic diseases such as IPF.

## INTRODUCTION

Cellular senescence and epigenetic alterations are among the hall markers of aging [1], and they are interconnected contributors to age-related diseases, including idiopathic pulmonary fibrosis (IPF) [2]. Cellular senescence is a state of permanent cell-cycle arrest and represents a complex and dynamic process. Initially, senescence was regarded as a protective mechanism to prevent damaged cells from proliferating and becoming cancerous [3]. However, emerging evidence shows that aging is accompanied by the accumulating senescent cells, these senescent and near senescent cells contribute significantly to aging and age-related diseases [4].

IPF is a prototypical age-related disease [5]. Transforming growth factor – β (TGF-β1) is a central cytokine that drives lung fibrosis, with fibroblasts being one of its major targets [6]. TGF-β1 induces the differentiation of lung fibroblast into myofibroblast, characterized by the increased expression of α-smooth muscle actin (α-SMA), and enhanced production of extracellular matrix, such as collagen [7]. TGF-β1 signaling pathways are critical for lung fibroblast differentiate and collagen synthesis, involving both SAMD-dependent and SMAD-independent pathways, such as the p38-mito-activared protein kinase (MAPK) pathway [8]. Numerous studies have reported the essential role of TGF-β1 signaling in the development of lung fibrosis. Additionally, IPF fibroblasts exhibit altered TGF-β-regulated pathways [9] and increased cellular senescence [10], which would reinforce profibrotic signaling and exacerbate disease progress [9, 11].

Although cellular senescence is recognized as a major driver for aging, the underlying mechanisms remain poorly understood. A variety of factors can induce senescence, including oxidative stress, telomere shortening, as well as epigenetic dysregulation [3]. These stressors contribute to DNA damage and interfere with DNA replication, disturb replicative DNA synthesis and replication fork stability, ultimately promoting the senescent phenotype [12]. The transition from a proliferative to a senescent state requires the transcriptional regulation of numerous genes, a process tightly controlled by epigenetic mechanisms [13]. Among these, post-translational histone modifications, which regulate chromatin architecture, play a key role in modulating gene expression [13]. For example, H4K16 acetylation (H4K16ac) promotes chromatin relaxation and is associated with active transcription [14]. H4K16ac has been linked to both aging and age-related diseases, including IPF [15–17], and has been reported to regulate the expression of α-SMA and collagen expression in primary lung fibroblasts derived from IPF patients [17].

Previously, we reported differential levels of H4K16ac in young low population doubling (LPDL) and near-senescent/senescent high population doubling (HPDL) lung fibroblasts, which contribute to the apoptosis resistance associated with cellular senescence [18]. In this study, we examined the differential responses of young (LPDL) and near-senescent/senescent (HPDL) lung fibroblasts to TGF- β1 and explored the epigenetic mechanisms contributing to the fibroblast activation in fibrosis.

## METHODS

### Cell culture and treatment

IMR90 human lung fibroblasts were purchased from Coriell Institute for Medical Research (Camden, NJ, USA). Cells were cultured in Dulbecco's modified Eagle's medium (DMEM, Life Technologies, Grand Island, NY, USA) with 10% fetal bovine serum (FBS, Life Technologies) with 5% CO_2_ at 37° C. The cell population doubling was calculated at each time of passage with formula PDL=3.32 [log (total viable cells at harvest/total viable cells at seed)]. Usually, cells with PDL <30 were categorized as low PDL (LPDL; or as young), and PDL above 40 as high PDL (HPDL; or as near senescent/senescent). As a note, in this study HPDL cells include both senescent and near-senescent fibroblasts, with the latter retaining proliferative capacity but at a much lower rate than young LPDL cells. Primary IPF lung fibroblasts were obtained from surgical explants as previously described [19], used before passage five, and donor information is provided in Supplementary Table 1. IPF diagnoses were made by clinicians blinded to the studies according to the American Thoracic Society/European Respiratory Society guidelines [20]. Cells were seeded at 2x10^6^ in 100 mm dish, when at 70% confluent, the media was changed to serum free for overnight. The TGF-β1 at 2ng/ml was added next day, or 2 hours after the inhibitor was added; only vehicle was added for control. The cells were collected at different time points as indicated in the text after adding TGF-β1. The p-38 MAPK inhibitor SB202190 (Cell Signaling, Danvers, MA, USA) was added at 10 μM according to previously published studies [21], alone or 2-hour before adding TGF-β1.

### Antibodies and immunoblotting

Antibodies used for immunoblotting are against p16 (#18769), p38 (#9212) recognizes p38α, β, or γ, phorspho-p38 (#9211) recognizes Thr180/Tyr182 of p38, phorspho-SMAD3 (#9520), SMAD (#9523), β-tubulin (#2128) or β-actin (#3700) all from Cell Signaling (Beverly, MA, USA). Antibodies against α-smooth muscle actin (#03-61001) was from American Research Products (ARP, Waltham, MA, USA), anti-Col3A1 (A3795) was from Abclonal (Woburn, MA, USA). For antibodies against H4K16ac and H4, and H4K16ac used for ChIP assays were from Active Motif (Carlsbad, CA, USA).

For western blot, the protein from whole cell lysate was collected and the concentration was measured by a Micro BCA protein assay kit (Thermo Fisher Scientific, Grand Island, NY, USA). The nuclear proteins were extracted by using the EpiQuick Nuclear extraction kit (Epigentek, Brooklyn. NY, USA). The analyzation of western blots was carried out as described before [22]. The normalization was performed by stripping the membrane after probing for the protein of interest, and then re-probing with the control protein. Total histone H4 was used as loading controls for nuclear extracts.

### Quantitative real-time RT-PCR

The total RNA was collected at the time indicated in the text, with a RNeasy kit (Qiagen, Valencia, CA, USA), then transcribed to cDNA with a reverse transcription cDNA synthesis kit (Clontech, Mountain View, CA, USA). The quantitative real-time RT-PCR were carried out with SYBR green mix in triplicates and normalize to β-actin using the ΔΔCt method. The primers for α-SMA are: F: 5′-TCCTCATCCTCCCTTGAGAA-3′, R: 5′-ATGAAGGATGGCTGGAACAG-3′; for Col3A1 are: F: 5’- -ATTGCCTGGGATCACTGGAGCAC -3’ and R: 5’- CTGGTTTCCCACTTTCACCCTTG-3’; for β-actin are F: 5’- TGCTATCCAGGCTGTGCTAT-3’, and R: 5’- AGTCCATCACGATGCCAGT-3’.

### Chromatin immunoprecipitation assays

Chromatin immunoprecipitation (ChIP) assays were performed as per manufacturer's protocol (Epigentek, Brooklyn, NY), with minor modifications [23]. ChIP-DNA was amplified by real-time PCR with primers of the following: the primers for α-SMA ChIP are: F: 5’-GAGGTCCCTATATGGTTGTGTTAG-3’, and R: 5’-AGCTGAAAGCTGAAGGGTTAT-3’; for Col3A1 ChIP primer set, F: 5’-CACACATAAAGCCGCACAAC -3’, R: 5’-GAGCTTGAGAGAGATGCACAA-3’. Results are normalized to input DNA.

### Statistical analysis

Statistical analysis was performed using Student’s *t* test or one-way ANOVA statistical analyses. Data are expressed as mean ± standard error (SE). The analysis was done using GraphPad Prism 10.4.1 for Windows, GraphPad Software (San Diego, CA, USA). Statistical significance was defined at *p* < 0.05. Densitometric analyses of Western blots were performed using the public domain NIH Image program of Image J.

### Data availability

The data that supports the findings of this study are available from the corresponding author upon reasonable request.

## RESULTS

### Similar TGF-β1 induced upregulation of profibrotic genes in young LPDL and near senescent/senescent HPDL lung fibroblasts

**Figure 1:**
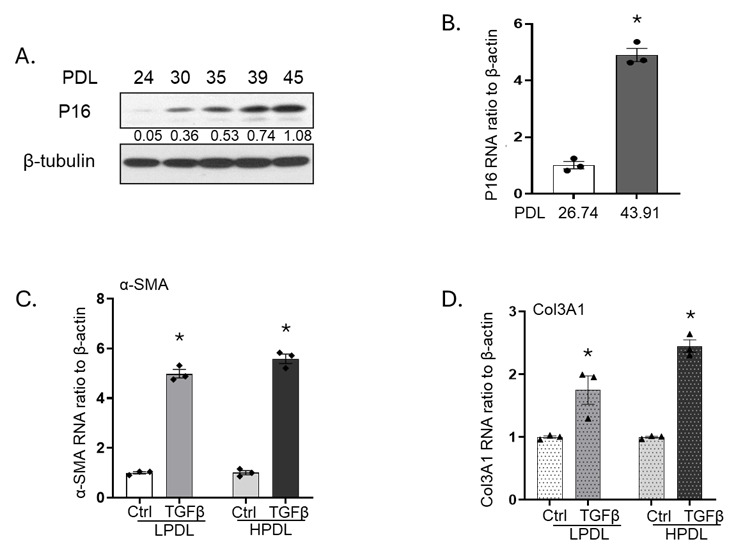
**Characterization and responses to TGF-β1 in IMR90 human diploid lung fibroblasts.** (**A**) p16 expression levels of IMR90 lung fibroblasts at the indicated population doubling levels (PDL). β-tubulin was used as loading control. Numeric values indicate the densitometric ratio of p16 to its corresponding β-tubulin. (**B**) p16 mRNA levels in LPDL or HPDL fibroblasts at baseline, measured by real-time RT-PCR and normalized to β-actin. (**C**, **D**). α-SMA (**C**) and Col3A1 (**D**) mRNA levels in LPDL and HPDL cells treated with or without TGF-β1 (2ng/ml) for 24 hours. Data are normalized to β-actin and represent the average of at least three independent experiments. Bar graphs indicate mean ± standard errors (SE). * p<0.05, compared with the control in the same group.

Cellular senescence plays an important role in lung fibrosis. In this study, senescence was induced by serial passaging of human lung fibroblast IMR90. The phenotype characterized as high population doubling (HPDL) has been reported before [18] of PDL 40 and above, includes both senescent cells and near senescent cells that retain proliferative capacity but divide much slower than young cells. Young lung fibroblasts were defined as low population doubling (LPDL) with PDL under 30. As shown in Figure 1A, the fibroblasts doubling level from 24 to 45 showed an increasingly intensification of p16, indicating the phenotype of the HPDL cells are getting senescent (Figure 1A). We further confirmed the expression of p16 at RNA level by quantitative real-time PCR, the PDL 43.91 showed significantly higher expression than the PDL of 26.74 cells (Figure 1B).

TGF-β1 is a well-known profibrotic cytokine in fibrotic related diseases, including pulmonary fibrosis [9]. We and others have reported increased profibrotic genes, like α-smooth muscle actin (α-SMA), collagen 3A1 (Col3A1) in response to TGF-β1 [24, 25]. Due to the significantly different phenotypes of the young and senescent cells [26], we examined if there are differences in gene expression in response to TGF-β1 in IMR90 cells of LPDL and HPDL. Despite the higher baseline expression of the HPDL cells of the two genes (Supplementary Figure 1), our data showed that both LPDL and HPDL cells have similar patterns of up-regulated α-SMA after 24 hours of TGF-β1 treatment (Figure 1C). Col3A1 is reported to be increased in hyperoxia-induced senescent lung fibroblasts [27], in our model of replicative induced senescence, the Col3A1 RNA expression showed similar upregulation as α-SMA (Figure 1D). Overall, although the young LPDL and near senescent/senescent HPDL fibroblasts are phenotypically different, their response to TGF-β1 induced up-regulation of profibrotic genes α-SMA and Col3A1 are similar.

### p-38MAPK pathway in LPDL and HPDL lung fibroblasts in response to TGF-β1

**Figure 2:**
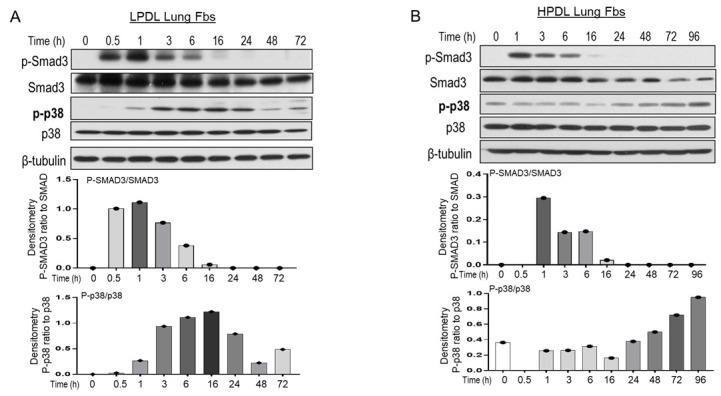
**IMR90 LPDL or HPDL cell response to TGF-β1-induced signal pathways.** (**A**, **B**) LPDL (**A**) and HPDL (**B**) lung fibroblasts lysates were collected at the indicated time points after treatment with TGF-β1 at 2ng/ml. β-tubulin was used as a loading control. Bar graphs was calculated with the shown western blots densitometric ratio of p-SMAD3 to SMAD3, or p-p38 to p38 in LPDL (**A**) or HPDL (**B**) lung fibroblasts. Similar western blots of LPDL and HPDL in response to TGF-β1 were in Supplementary Figure 2, which densitometry of p-p38 to p-38 was shown as averaged for 3 independent repeats (Supplementary Figure 2).

To explore the underlying response to TGF-β1 in LPDL and HPDL lung fibroblasts, we did a time course response in these cells. The collected whole cell lysate started as early as 0.5- or 1-hour till as late as 72- or 96- hours (Figure 2). In both LPDL and HPDL fibroblasts, there is a peak of the canonical pathway p-SMAD3 at 1 hour, which decreased and almost disappeared at 16 hours after adding TGF-β1, showing similar pattern in LPDL and HPLD cells of this pathway (Figure 2). In the SMAD independent p38-MAPK pathway, we noticed an earlier upregulation of phorspho-p38 MAPK in young LPDL lung fibroblasts, with increasing tense to 24-hour, and then reduced at later time points like 48 or 72 hours (Figure 2A). On the other hand, for the near senescent/senescent HPDL cells, the response to TGF-β1 is slow but sustained. There is no clear peak noticed at early time points of phorspho-p38 MAPK pathway in HPDL, which the pathway slowly intensifies after almost 24 or 48 hours. Although not as robust as LPDL cells, the pathway sustained after adding TGF-β1 till 72 or 96 hours (Figure 2B, additional figure at online Supplementary Figure 2 with densitometry average of 3 repeats).

Because α-SMA upregulation is a well-established marker of myofibroblast differentiation in lung fibrosis [28], many studies have shown that it increases 24 h after TGF-β1 treatment [29, 30]. We further confirmed and compared the α-SMA expression in LPDL and HPDL cells at different time points. Both cell types showed clear upregulation at 24h (online data, Supplementary Figure 3), which was the time point selected for most subsequent experiments. In summary, in response to TGF-β1 treatment, the p-SMAD3 pathway displayed a similar timing and duration of the peaks in IMR90 LPDL and HPDL lung fibroblasts. However, the p-p38 MAPK pathway appears to be quicker and more robust in young LPDL cells, whereas near senescent/senescent HPDL cells exhibited a slower but more sustained response.

### Up-regulated pro-fibrotic proteins induced by TGF-β1 are blocked by p38-MAPK inhibitor

**Figure 3:**
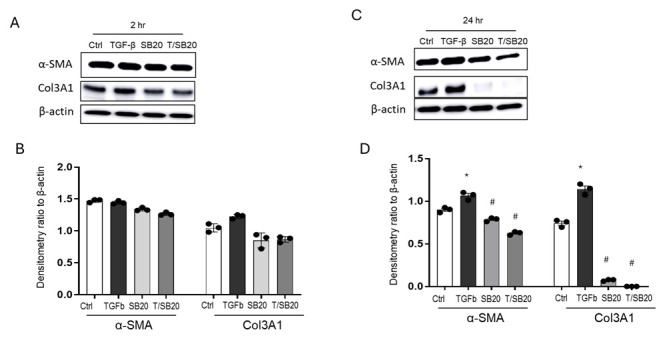
**α-SMA and Col3A1 protein expression in response to TGF-β1 with or without p38 MAPK inhibitor SB202190.** IMR90 cells were subjected to p38 MAPK inhibitor alone or, treated with TGF-β1 (2ng/ml) at 2h (**A**, **B**) or 24h (**C**, **D**) for densitometry with or without pretreatment for 2h of SB202190 (10μM). β-actin was used as a loading control. T indicates TGF-β1; SB20, SB202190. Results are average of at least three independent experiments. Bar graphs indicate mean ± standard errors (SE). * p<0.05, compared to the vehicle control (Ctrl) within the same group, #p<0.05 compared to TGF-β1 only group.

Due to the observed differences in p-38 MAPK in young LPDL and near senescent/senescent HPDL cells in response to TGF-β1 (Figure 2), we decided to block the p-38 MAPK pathway with its specific inhibitor and examine the effects of such blocking on fibrotic markers. Because of the similar pattern of responses to TGF-β1 at 24h, and the robust response of LPDL cells, to simplify, we used LPDL cells to explore whether p-38 MAPK inhibition will alter α-SMA and Col3A1 expression at protein levels. We treated the cells with p-38 MAPK inhibitor SB202190 according to previously published studies [21], alone or 2 hours before adding TGF-β1 in the culture. The cell lysate was collected at 2 or 24 hours after adding TGF-β1. At 2 hours after adding TGF-β1, there is not much notable differences (Figure 3A, 3B). At 24 hours, the inhibitor treated groups showed significantly reduced α-SMA and Col3A1 at protein levels in response to TGF-β1 (Figure 3C, 3D). This shows that p-38 MPAK inhibition reduces lung fibroblast differentiation partially by reducing α-SMA and Col3A1 at protein levels.

### p-38 MAPK inhibition downregulates the transcription of α-SMA and Col3A1 in myo-fibroblasts

**Figure 4:**
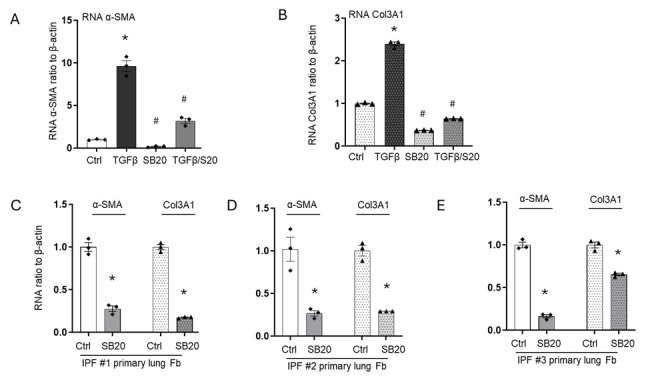
**Transcriptional levels of α-SMA or Col3A1 in lung fibroblasts with or without p38 MAPK inhibitor.** (**A**, **B**) α-SMA (**A**) or Col3A1 (**B**) mRNA were checked in IMR90 cells with SB202190 (SB20) alone, or 2 h before TGF-β1 treatment at 2ng/ml, all collected after 24 hours of adding TGF- β1. mRNA expression was measured by real-time RT-PCR and normalize to β-actin. (**C**–**E**) Primary lung fibroblasts from three different IPF patients were treated with SB202190 for 24h, followed by RNA collection and measurement of α-SMA and Col3A1 mRNA expression by real-time RT-PCR, normalized to β-actin. Results are averages of at least three independent experiments. Bar graphs indicate mean ± standard errors (SE). * p<0.05, compared to the vehicle control (Ctrl) within the same group; # p<0.05 compared to TGF-β1 treated group.

Next, we examined the transcription level changes of α-SMA and Col3A1 in the myofibroblasts either by TGF-β1 treated IMR90 or in primary IPF cells with p-38 MPAK inhibition. We measured the RNA expression levels of α-SMA and Col3A1at 24 hours after TGF-β1 treatment with or without p-38 MAPK inhibition as the same conditions shown in Figure 3C. The up-regulated α-SMA (Figure 4A) and Col3A1 (Figure 4B) by TGF-β1 are significantly reduced by p-38 MAPK inhibition. In addition, adding SB202190 alone also significantly reduced the transcription level of α-SMA and Col3A1 (Figure 4A, 4B).

Prolonged activation of myofibroblasts is a critical feature in IPF, characterized by upregulated α-SMA and collagen [7]. Therefore, we further examine whether p38 MAPK inhibition could reduce fibrotic markers in the primary IPF myofibroblasts. Three different primary IPF myofibroblast cultures were treated with the inhibitor SB202190 for 24 hours, all of which showed significant downregulation of α-SMA and Col3A1 mRNA (Figure 4C–4E). Due to the well-known heterogeneity of IPF primary cells, the degree of sensitivity varied, particularly for Col3A1, with one culture showed nearly 80% reduction (Figure 4C) and another only about 40% (Figure 4E). Although the causes of this heterogeneity are unclear, its potential relevance to personized medicine makes it important for future investigations. Nonetheless, our data demonstrates that p38 MAPK inhibition reduces the transcription of the profibrotic genes α-SMA and Col3A1 in myofibroblasts.

### p-38 MAPK inhibition affects histone modification H4K16ac in lung fibroblasts

**Figure 5:**
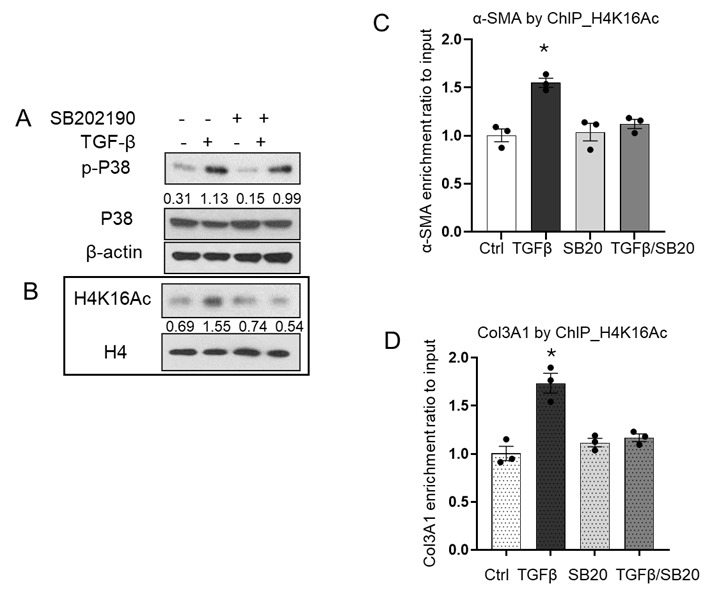
**Effects of TGF-β1 and p38 MAPK inhibition on H4K16ac.** (**A**) p-p38 MAPK levels in IMR90 24h after treatment with TGF-β1 (2ng/ml) with or without 2h pretreatment with SB202190, or with inhibitor alone. Whole cell lysates were prepared; β-actin was used as a loading control. (**B**) Nuclear extracts from cells prepared as in (**A**) were subjected to western blotting. H4 was used as a loading control. Numbers indicate densitometric ratio of p-p38 to total p38, or H4K16ac to H4. (**C**, **D**) ChIP assays with H4K16ac pulldown showing association with α-SMA (**C**), and Col3A1 (**D**) promoter regions. Quantitative ChIP assays were performed to analyze the association of H4K16ac with α-SMA or Col3A1 at the conditions indicated in A. DNA were crosslinked with the immunoprecipitated protein using specific antibody H4K16ac. Quantitative PCR was analyzed using 2^−ΔΔCt^ method, with results normalized to input DNA relative to vehicle control (Ctrl). Bar graphs represent mean ± SE from the average of at least three independent experiments; ^*^p<0.05 compared to vehicle control.

IPF is an age-related disease, and cellular senescence has emerged as a critical mechanism in its patho-genesis [4, 31]. The age-associated histone modification, H4K16ac has also been implicated in this process [17, 18]. Previously, we reported that H4K16ac levels are reduced in HPDL cells [18] as well as in lung fibroblasts from aged mice [17]. We also found that H4K16ac levels are elevated in primary IPF lung fibroblasts and in lung fibroblasts from aged mice with bleomycin-induced lung fibrosis compared to their respective controls [17]. In the present study, we examined changes in H4K16ac in our cellular model of replication-induced senescence and assessed whether H4K16ac is associated with α-SMA and Col3A1 gene expression. Given that many IPF fibroblasts are senescent or near senescent [4, 31], we evaluated H4K16ac responses in near senescent/senescent HPDL cells following TGF-β1 stimulation. Phosphorylated p38 MAPK was upregulated 24h after TGF-β1 treatment, and this upregulation was attenuated by pre-treatment with the inhibitor SB202190 (Figure 5A). Correspondingly, TGF-β1 induced increases in global H4K16ac levels were reduced by p38 MAPK inhibition (Figure 5B, and online data Supplementary Figure 4 for siRNA p38 inhibition). A similar pattern was observed in primary IPF lung fibroblasts treated with TGF-β1 and p38 MAPK inhibitor (Online data Supplementary Figure 4).

As we have demonstrated before, the global levels of histone modifications do not necessarily reflect their levels at specific gene loci [18, 32]. Therefore, we performed ChIP assays to examine potential changes in H4K16ac enrichment at the promoter regions of α-SMA and Col3A1. In response to TGF-β1, both α-SMA (Figure 5C) and Col3A1 (Figure 5D) showed increased H4K16ac enrichment at their promoter regions. This enrichment was reduced by p38 MAPK inhibition alone or by pretreatment with the inhibitor followed with TGF-β1 stimulation (Figure 5C, 5D). Overall, our data indicate that p38 MAPK mediates fibroblast activation by up-regulating the profibrotic genes α-SMA and Col3A1, at least in part through increased enrichment of the active histone mark H4K16ac at their promoter regions.

## DISCUSSION

Histone modification and cellular senescence play a critical role in age-related diseases, such as IPF. In this study, we examined the responses to TGF-β1 in IMR90 lung fibroblasts with replication-induced HPDL (including proliferating near-senescent and non-proliferating senescent cells) and LPDL (mostly young, actively proliferating cells). Although both HPDL and LPDL fibroblasts exhibited similar SMAD pathway activation and upregulation of the profibrotic genes α-SMA and Col3A1 in response to TGF-β1, HPDL fibroblasts showed a slower but more sustained p-p38 MAPK pathway compared to LPDL cells. Inhibition of the p38 MAPK pathway reduced α-SMA and Col3A1 expression in both HPDL and LPDL fibroblasts upon TGF-β1 stimulation, as well as in primary lung fibroblasts derived from IPF patients. This reduction in gene expression was likely mediated by decreased H4K16ac enrichment at the promoter region of these genes. Our findings are consistent with previous reports that p38 inhibition slows IPF progression, possibly by attenuating prolonged myofibroblast activation in lung fibrosis.

Cellular senescence was first reported in the early 1960s [33], and has since been linked to aging [34, 35]. Senescence contributes to various physiological conditions, including embryonic development and tissue patterning [36]. It also plays protective roles, such as preventing the proliferation of damaged cells that could become cancerous and promoting tissue regeneration during wound healing by recruiting immune cells to eliminate senescent cells [37]. Despite these beneficial roles, senescence is implicated in many age-related diseases, including chronic lung diseases, such as IPF [2]. The incidence of IPF increases significantly with age, particularly in association with the accumulation of senescent cells in the lung [38]. Emerging evidence indicates that IPF lung fibroblasts demonstrate a senescent phenotype [39]. *In vitro* studies are commonly performed with low-passage cell lines; however, our current and previous studies have shown that cells with low doubling levels display different phenotype from the cells with high doubling levels [18] and may respond differently to cytokines such as TGF-β1. Although we observed similar upregulation of profibrotic genes in response to TGF-β1 in both low and high PDL cells, the activation patterns of the p38 MAPK pathway seems different. This differential signaling in HPDL may contribute to the prolonged activation of fibroblasts in age-related fibrotic diseases, similar to a previous study showing that IPF fibroblasts maintain elevated levels of phosphorylated STAT3 [11].

TGF-β1 is a central profibrotic cytokine that mediates fibroblast differentiation, primarily through the well-characterized SMAD-dependent path-way [40]. In addition, the non-canonical SMAD-independent pathways, including p38MAPK, are also critical for fibroblasts activation [9]. Numerous studies have reported that p38 inhibition alleviates bleomycin-induced lung fibrosis in animal models [41-44], involving effects on macrophages, epithelial cells, and fibroblasts. In a mice model with a dominant-negative p38 mutation in alveolar epithelial type II cells, the animals carrying the mutation developed less severe and extensive fibrosis compared to those in the wildtype controls [43].

Pirfenidone, a p38 inhibitor, was the first drug approved for treatment of IPF in Europe in 2011 [45], and then in the U.S. in 2014 [46]. While Pirfenidone has been show to slow IPF progression, its exact mechanisms of action remain unclear, though likely involving inhibition of excessive fibrotic responses [47]. p38 MAPK is a critical signaling pathway involved in various cellular processes. We previously reported that p38 MAPK mediates the TGF-β1–induced downregulation of the anti-fibrotic gene caveolin-1 (Cav-1) in differentiated myofibroblasts [30]. In that study, cells harboring a mutant p38 MAPK failed to downregulate Cav-1 in response to TGF-β1 and exhibited a less pronounced upregulation of α-SMA, a pattern similar to what we observed here using a p38 MAPK inhibitor in both LPDL and HPDL fibroblasts.

The p38 MAPK pathway is also implicated in other fibrosis-related processes, including promotion of inflammation, induction of epithelial-to-mesenchymal transition, as well as myofibroblast differentiation [48]. In this study, we focused specifically on fibroblast differentiation in response to TGF-β1. Persistent and uncontrolled activation of lung fibroblasts is a key driver of fibrotic disease progression, including lung fibrosis [49], although the underlying mechanisms remain incompletely understood. While the SMAD pathway is typically the primary mediator of fibroblast differentiation, our results showed that phosphorylated SMAD3 responded similarly and transiently in both LPDL and HPDL cells. In contrast, near senescent/senescent HPDL fibroblasts exhibited slower and more sustained activation of phosphorylated p38 MAPK compared to the young LPDL fibroblasts. Inhibition of p38 MAPK reduced profibrotic genes expression in both LPDL and HPDL cells, consistent with prior studies showing that pirfenidone attenuates fibroblast responses to TGF-β1 [50]. However, previous studies did not investigate age-related differences in p38 pathway activation in lung fibroblasts. These findings align with prior studies showing that p38 MAPK inhibition ameliorates lung fibrosis in animal models [41, 42], although some of those studies focused on anti-inflammatory mechanisms. Furthermore, we examined whether p38 MAPK inhibition affects epigenetic regulation through histone acetylation.

H4K16ac is a histone mark associated with active transcription and has been implicated in the patho-genesis of lung fibrosis [17]. TGF-β1 related signaling modulates histone acetylation through downstream effectors, likely by influencing enzymes that maintain the balance of histone modifications [51]. Although we did not assess the expression or activity of H4K16ac related enzymes in this study, we observed increased global H4K16ac levels in both IMR90 and IPF lung fibroblasts after TGF-β1 stimulation. Similar changes in specific histone acetylation marks have been reported in renal fibroblasts exposed to TGF-β1 [51]. Combined with our previous observation that global histone modification changes can differ from those at specific gene loci [18], this prompted us to investigate whether H4K16ac is involved in regulating α-SMA and Col3A1. We found that TGF-β1 treatment enriched H4K16ac at the promoter region of α-SMA and Col3A1, and inhibition of p38 MAPK markedly reduced this enrichment. This reduction was accompanied by decreased mRNA expression of these genes, suggesting that p38 MAPK regulates their transcription by modulating H4K16ac occupancy at their promoters. Similar p38 MAPK–mediated histone modification changes have been reported in other contexts. For example, inhibition of p38 MAPK reduced H4K16ac levels in both inner and outer cells during early blastocyst development [52]. In addition, p38 MAPK has been shown to mediate UV radiation–induced histone H3 acetylation, chromatin relaxation, and histone H3 serine 10 phosphorylation [53]. Together, these findings identify p38 MAPK as an important regulator of histone modifications and fibroblast activation in lung fibrosis. Our results suggest that p38 MAPK contributes to the trans-criptional regulation of α-SMA and Col3A1 at least by influencing H4K16ac binding at their promoters. Whether the observed reduction of H4K16ac is due to decreased acetylation or increased deacetylation remains to be determined.

Overall, our study demonstrated the responses of young LPDL and near senescent / senescent HPDL lung fibroblasts to TGF-β1. We show that TGF-β1 increases H4K16ac enrichment at the promoters of α-SMA and Col3A1, leading to their up-regulation. This effect is blocked by p38MAPK inhibition, resulting in reduced expression of these profibrotic genes. The near senescent/senescent HPDL fibroblasts exhibit a more sustained activation of the p38 MAPK pathway in response to TGF-β1, which may likely contribute to the prolonged activation of myofibroblasts in pulmonary fibrosis. Inhibiting this pathway could therefore disrupt fibroblast activation, likely through epigenetic regulation by H4K16ac. Our findings provide further support of targeting p38 MAPK as a potential therapeutic strategy for age-related fibrotic diseases, such as IPF.

## Supplementary Material

Supplementary Figures

Supplementary Table 1
