# Supplementary material for: P38 MAPK is involved in epigenetic regulation of fibrotic genes in replication induced senescence in lung fibroblasts
Source: Aging (Albany NY). 2026 Mar 3;18(1):67–81. doi: 10.18632/aging.206357 (PMC13285948; doi:10.18632/aging.206357)
Supplement: Supplementary Figures [file aging-18-1-206357-s001.pdf]

SUPPLEMENTARY FIGURES

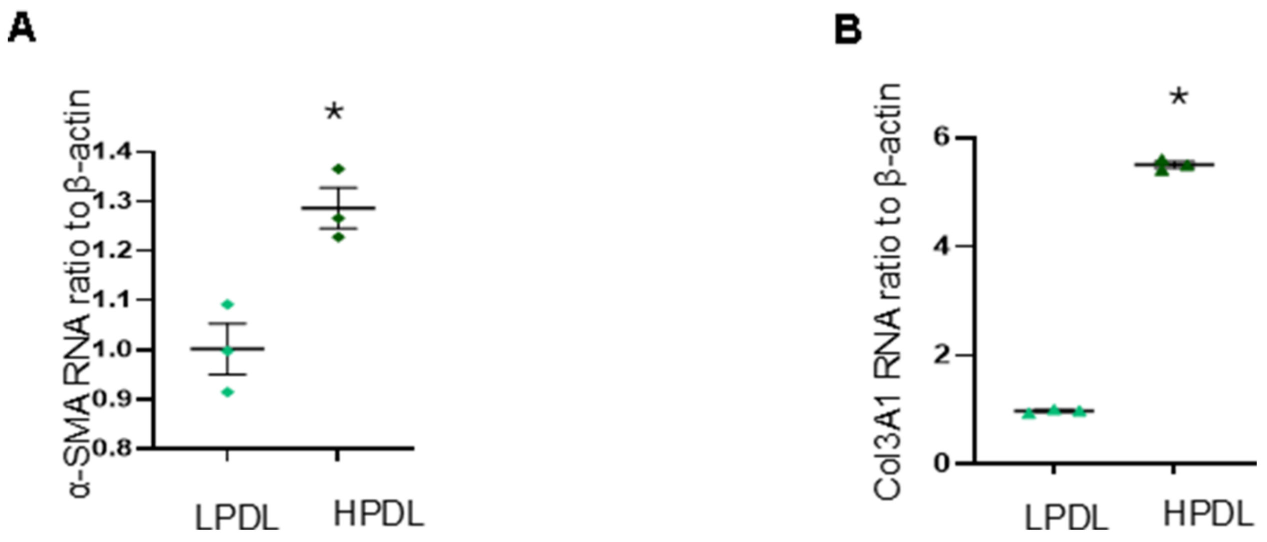

Supplementary Figure 1. Real-time RT-PCR of baseline expression of α-SMA (**A**) and Col3A1 (**B**) in LPDL or HPDL. Results are averages of at least three independent experiments. Data shown as mean ± standard errors (SE). * p<0.05, compared to the LPDL in its own group.

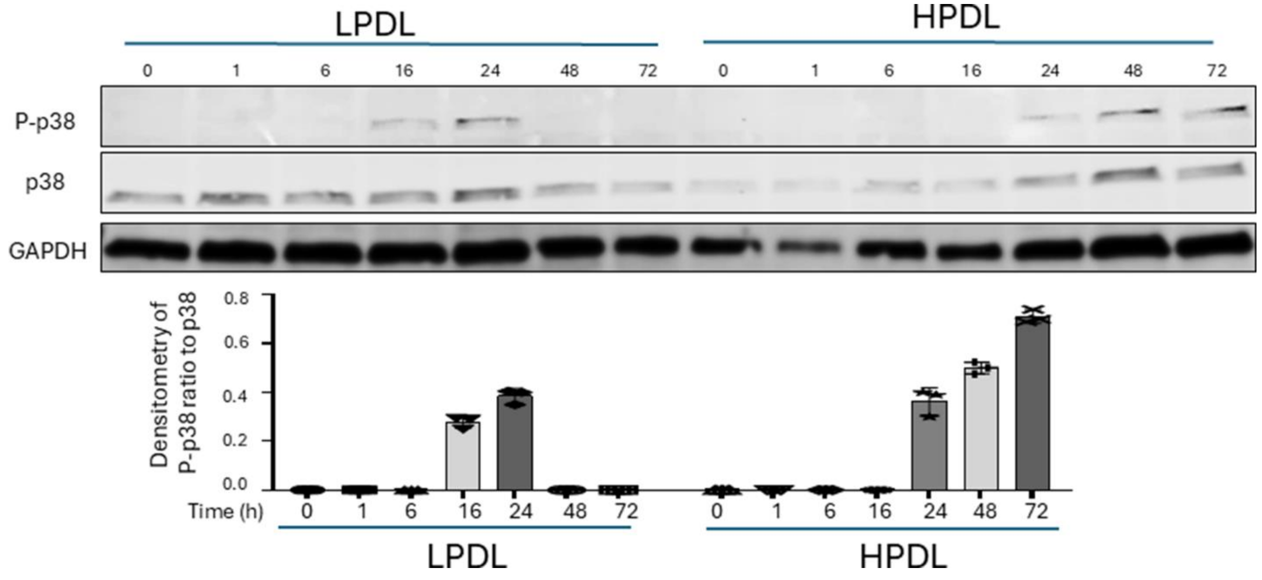

Supplementary Figure 2. IMR90 LPDL and HPDL cells response to TGF-β1 induced p-p38 and p38 signal pathway. The cell lysate was collected at the time points after adding TGF-β1 at 2ng/ml. GAPDH was the loading control. Bar graphics represents densitometry of p-p38 to p38 from mean± standard error of three independent repeats.

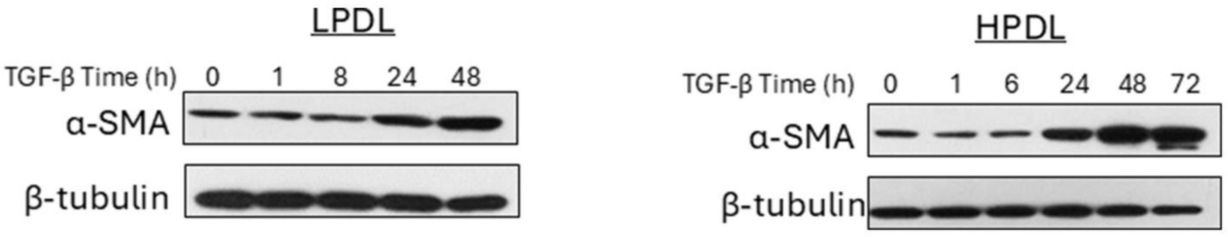

Supplementary Figure 3. LPDL (**A**) or HPDL (**B**) in response to TGF-β at the time points indicated, the expression change of α-SMA was examined. The β-tubulin is the loading control.

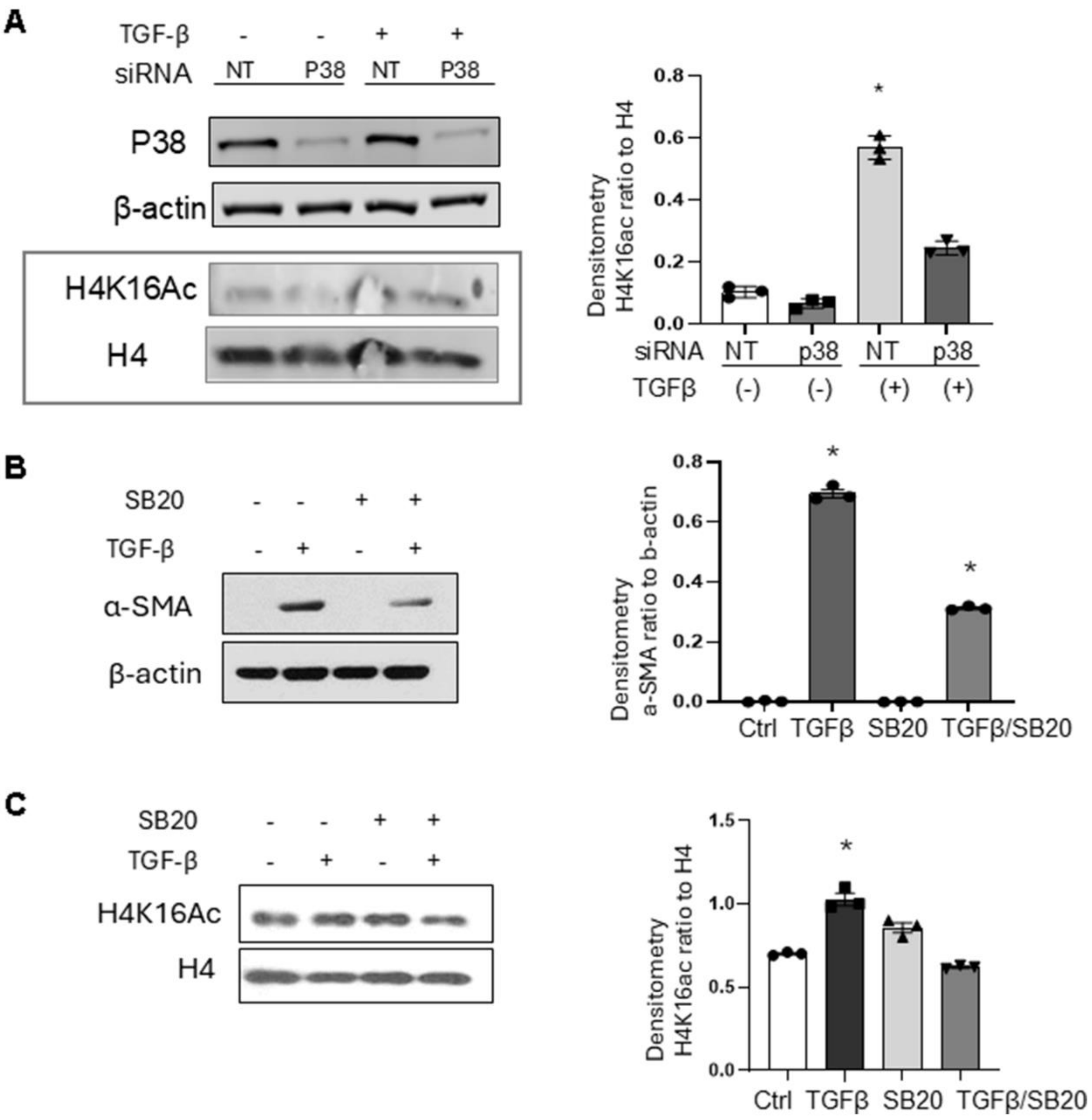

Supplementary Figure 4. Lung fibroblasts transfected with siRNA p38 (sc-29433 Sant Cruz Biotechnology) were treated with TGF-β1 the same ways as in Figure 5, but without the inhibitor to further confirm the changes of global H4K16ac levels (**A**). Primary IPF lung fibroblasts were subjected to TGF-β1, with or without the p38 MAPK inhibitor SB203580 as in Figure 5 A, B. Cells were collected after 24h, and α-SMA protein levels were assessed by western blots (**B**). Changes in global H4K16ac levels in IPF cells were examined in nuclear extracts by western blots (**C**). Histogram represents the densitometric ratio of H4K16ac to H4, or α-SMA to β-actin, shown as the mean ± standard error (SE) of three independent experiments. *$p < 0.05$ compared with control (Ctrl) in the same group.
