# Supplementary material for: P38 MAPK is involved in epigenetic regulation of fibrotic genes in replication induced senescence in lung fibroblasts
Source: Aging (Albany NY). 2026 Mar 3;18(1):67–81. doi: 10.18632/aging.206357 (PMC13285948; doi:10.18632/aging.206357)
Supplement: Supplementary Table 1 [file aging-18-1-206357-s002.pdf]

SUPPLEMENTARY TABLE

Supplementary Table 1. Donor information for the IPF primary lung fibroblasts used in this study.

ID	Age/Race/Gender	Smoke-history	Diagnosis
#1	70/white/male	Never smoked	IPF
#2	69/white/male	Past smoker	IPF
#3	74/white/male	Never smoked	IPF
